# International collaboration between low‐middle‐income and high‐income institutions to improve radiation therapy care delivery

**DOI:** 10.1002/acm2.14306

**Published:** 2024-02-23

**Authors:** Diana Ng, Tatiana De Martin, Po Ting Wu, Peter Huang, Jennifer Davis, Jamil Serdiña, Jamie Nash, Sarah Knighton, Sherwin Cala, Tisha Lee V. Eduave, Anthony Albert Abad

**Affiliations:** ^1^ GenesisCare Solutions Alexandria Australia; ^2^ Philippine Oncology Center Corporation Quezon City Philippines

**Keywords:** low‐middle income institutions, radiation therapy, radiotherapy infrastructure

## Abstract

**Introduction:**

The Philippines is a lower‐middle‐income island country with over 153 000 new cancer diagnosis each year. Despite many patients needing radiotherapy as part of disease management, there remains limitations to access. Currently, the Philippines has 50 linear accelerator facilities serving a population of 110 million. However, given the recommendation of 1 linear accelerator for every 250 thousand people, it is evident that the demand for accessible radiotherapy resources is significantly underserved in the country. This paper outlines the collaboration between GenesisCare Solutions (GCS) and Fairview Cancer Center (FCC) to address efficiency and access within the radiotherapy department at FCC.

**Methods:**

Through international collaboration between GCS and FCC, areas for improvement were identified and categorized into four domains: Dosimetry quality, Patient workflow, Data & Reporting, and Information Technology (IT) Infrastructure. Action plans were developed then implemented. A baseline measurement was obtained for each domain, and post‐implementation evaluation undertaken at 3 months, 6 months, and 12 months. Data captured within the electronic medical record system was extrapolated, and average treatment times were established for pre‐ and post‐engagement. A paired, 2‐tailed *t*‐test was used for statistical analysis of outcome parameters using IBM SPSS version 23 for all statistics.

**Results:**

Twelve months post‐initial engagement, all four domains saw positive outcomes. Improved plan quality linked to Intensity Modulated Radiotherapy (IMRT) utilization rates saw an increase from 20% to 54%. A significant reduction in patient average wait times was also observed, from 27 to 17 min (*p* ≤ 0.001). Prior to engagement, tracking patient demographics and diagnosis was not prioritized, post engagement an average of 92% diagnosis entry compliance was achieved.

**Conclusion:**

Through the collaboration of GCS and FCC, objectives in all action plan domains were achieved, highlighting the benefits of collaboration between low‐middle‐income and high‐income institutions.

## 1 INTRODUCTION

The Philippines^1^, ^2^, ^3^, ^4^, ^5^, ^6^ is a lower‐middle‐income island country with approximately 110 million people.^5^ Cancer is the country's third leading cause of morbidity and mortality.^7^ With over 153 000 new cancer diagnosis each year in the Philippines,^2^ the demand for radiotherapy access is ever‐increasing.

Radiotherapy (RT) is an essential element of a successful cancer care program, with approximately 50% of all cancer diagnosis patients benefiting from RT in the management of their disease.^8^ In 2020 the cancer diagnosis with the highest incidence and prevalence throughout the Philippines was breast cancer followed by lung and colorectal cancers.^2^, ^7^ All three diagnoses routinely include radiotherapy as an essential treatment component, either as the primary modality or as part of multimodal treatment. Currently, the Philippines has 50 linear accelerator (Linac) facilities for its population of 110 million,^3^, ^4^ with the recommendation of 1 linear accelerator for approximately 250 thousand population,^6^ It is evident that the demand for accessible radiotherapy resources is significantly underserved in the country.

Radiotherapy is one of the more cost‐effective treatment options for cancer management,^9^ however, due to significant outlay cost for facilities and equipment, there is a resultant limitation in access to treatment in most low‐ and middle‐income countries (LMICs)^9^, ^10^ as is seen in the Philippines. The Republic of Philippines Department of Health has pledged the Philippine Cancer Control Program to take steps to alleviate resource demand by providing patients greater access to care.^7^ The role of private practice in facilitating this increased access to quality radiotherapy is essential^11^ to not only further ease the burden on the public hospital system but also to further the research and development of radiotherapy within the Philippines.

Fairview Cancer Center (FCC) is a single Linac department located in metro Manila and is a part of the larger privately managed Philippine Oncology Center Corporation (POCC). GenesisCare Solutions (GCS) is a global oncology service provider offering a range of clinically led services that support the delivery of high‐quality and cost‐effective oncology care to patients. This paper outlines the collaboration between GCS and FCC to address efficiency and access within FCC's radiotherapy department.

## 2 METHODS

GCS began work with POCC in November of 2020, establishing a strong partnership with aims to enhance efficiency in the cancer patient's radiotherapy pathway at FCC. GCS functions as a service provider in the context of this initiative, offering essential services to meet the project objectives. The financial funding GCS receives is contingent upon the successful delivery of these services.

Through collaboration with the clinical team at FCC, priority areas for improvement within the clinic were identified. Clinical leaders at FCC were recruited to complete a comprehensive gap analysis survey identifying areas contributing to workflow inefficiencies and patient access limitations to establish a directed action plan addressing the needs of both clinical staff and patients. Areas of greatest opportunity were then categorized into four main domains to be targeted by the developed action plan: Dosimetry quality, Patient workflow, Data & Reporting, and Information Technology (IT) Infrastructure. Action plans for these four domains were developed then implemented to establish streamlined workflows, improve efficiency, and increase patient access to quality care.

As a result of the COVID‐19 pandemic, all interactions and engagement between GCS and FCC teams took place virtually via the Zoom online platform. This necessitated a strong reliance on continuous communication and the implementation of comprehensive supporting documentation for all introduced workflows and processes.

The need for improved dosimetry quality was evident in the feedback from FCC staff during the gap analysis survey. Factors contributing to dosimetry quality issues included tight time constraints and the need for additional staff training in modern planning techniques such as Intensity Modulated Radiotherapy (IMRT). This was reflected in the department's planning statistics, where only an estimated 20% of plans used an IMRT approach. To address immediate staffing challenges, remote dosimetry support from experienced planning staff in the GCS Australia team was proposed to alleviate the demanding workload on the local team while training and workflow transitions were introduced. Additionally, virtual education sessions were conducted once a week for the first 3 months of engagement with FCC planners and physicists. During these dosimetry sessions, the GCS dosimetry team would demonstrate planning a clinical plan using IMRT approach, providing justification for planning parameters, and developing planning templates within the planning system that would be used for future clinical cases. Additionally providing supporting educational material for both the dosimetry and treatment teams at FCC enabled further self‐directed learning and a point of reference for the local FCC teams. Introducing a templated approach to IMRT planning for disease sites such as prostate, colorectal, and head & neck cancers not only improved efficiency and alleviated time constraints but also maintained consistency with plan quality output. With the remote dosimetry support by the GCS team, as well as the dosimetry education sessions, a 60% IMRT utilization goal was set, lending to enhanced treatment quality. Monthly IMRT utilization was tracked through the oncology electronic medical record (EMR) system for each patient.

For patient workflow, a focus on reducing patient wait times was the priority. Prior to GCS engagement, patient wait times, defined as the time from patient check‐in at the department to treatment start time captured in the department's electronic medical system (EMR) averaged 27 min. This lent to limitations on the number of patients scheduled on the machine each day and impacted overall patient satisfaction with their treatment journey. Through the implementation of the electronic medical record system for patient scheduling, as well as introducing set patient appointment times, a goal of 20‐min wait times was established. These wait times were captured on an information dashboard and analyzed each month to identify any trends or improvements. Additionally, conducting remote training sessions for treatment staff to optimize daily patient setup processes, as well as treatment staff in‐services to establish a robust image‐matching process that supports a greater IMRT planning workload, further lent to safe efficient treatment workflows.

For Data & Reporting, universal data collection for patient treatment and treatment encounters was introduced using the department's EMR system. This was established as quality checklists (QCLs) under each patient file, to be completed by nursing and administration staff. These QCLs were designed by the GCS applications team in collaboration with FCC to capture relevant and informative data that was then used to provide key insights into patient demographics and outcomes as well as ensuring the patient experience was optimized.

For IT infrastructure, the shift to an electronic workflow from a paper‐based system was introduced. Prior to GCS engagement, the administration time consolidating paper records and manually recording daily treatment details for each patient was laborsome, and the physical storage and transport of all patient records each day was prone to error and inefficiencies. This transition to an electronic workflow required all clinical staff including administration, nursing, radiation oncologists, and the radiotherapy team to partake in training to upskill in proficient electronic record keeping. This not only allowed for greater operational efficiency and accuracy in the patient pathway, as seen through reduced patient wait times, but also lent to a greater capacity for meaningful data collection which will yield benefits for future research opportunities. Digital EMR checklists were designed and launched in the EMR system by the GCS applications team to capture succinct, relevant data at appropriate times in the patient treatment pathway. To support this change in IT workflow, additional EMR terminals were installed at multiple locations within the department.

The baseline outputs for IMRT utilization rate, patient wait times, and staff workflow compliance were gathered and then evaluated at 3 months, 6 months, and 12 months post‐implementation to evaluate the success of engagement as well as areas of potential improvement. Initially, daily virtual huddles with onsite staff were conducted to get direct and timely feedback on implementation and address any pinch points in the streamlined workflow. These daily huddles were reduced to three times a week then once a week as staff confidence and competency grew. This ensured clinical output and patient safety was maintained throughout go‐live.

A paired, 2‐tailed t test was used for statistical analysis of outcome parameters using IBM SPSS version 23 for all statistics. Data captured within the EMR system was extrapolated and average treatment times were established for pre and post‐engagement. Overall patient wait times were established from the 1^st^ quarter of engagement and compared to wait times from the 4^th^ quarter to track improvements since engagement.

## 3 RESULTS

Between January and December 2021, 225 patients were treated at FCC. Since the implementation of the electronic workflow and integrated data collection through this collaboration, systematic patient data that had not been tracked prior is now tracked for each patient attending the department. For all 225 patients, demographics and clinical variables were captured (Table 1). Most patients treated within the evaluation period were female, coming from the metropolitan Manila area with a mean age of 55 years. This data reflects the predicted disease demand for breast patients within the Philippines. This study was reviewed and approved by an internal review board (IRB).

**TABLE 1:** Patient demographic information over 12‐month period for 1 treatment machine as captured through the electronic workflow.

Variables	*N*	%
Age group		
21–20	9	4%
31–40	22	11%
41–50	53	23%
51–60	49	23%
61–70	55	25%
71–80	32	11%
81–90	5	2%
**Total**	225	
**Mean**	55	
**Median**	63	
Sex		
Female	146	65%
Male	79	35%
**Total**	225	
Place of residence		
Metro Manila	194	87%
Outside Metro Manila	29	13%
**Total**	223	

With the implementation of procedures geared to improve patient access and treatment quality; the following outcomes were observed.

Within the Dosimetry quality domain, since the introduction of remote planning support and planning up‐skill sessions through collaboration with GCS, the IMRT utilization for planning output achieved an average of 53.8% over a 12‐month period, up from an initial estimate of 20% output prior to GCS engagement. The most common patient diagnosis treated with IMRT was breast cancer (Table 2), specifically for chest‐wall and nodal involvement patients where an improvement in dosimetry quality is linked to an IMRT approach.^12^, ^13^

**TABLE 2:** Patient diagnosis information treated with an IMRT planning approach over 12‐month period for 1 treatment machine as captured through the electronic workflow.

Top 5 diagnosis		
Diagnosis	Patient count	%
Breast	52	20%
Palliative	33	13%
Cervix	30	12%
GI	17	7%
Endometrium	13	5%
Other	110	43%

**FIGURE 1:**
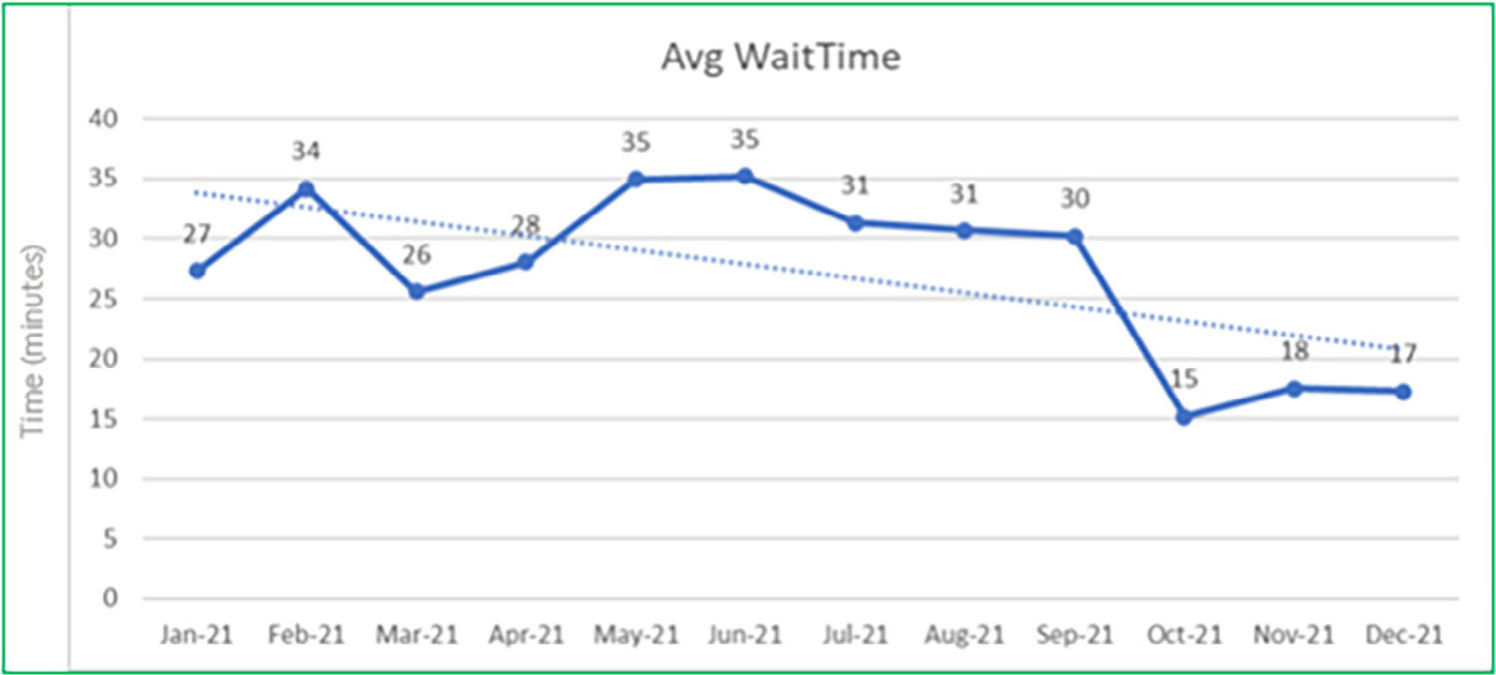
Average patient wait‐time over 12‐month period.

Positive outcomes were achieved within the patient workflow domain. The introduction of streamlined electronic workflows has lent to improved machine utilization, shorter patient wait‐times, and an overall improved patient experience. At the end of 12 months of data collection, a statistically significant reduction in average patient wait time was identified, with mean wait times reduced from 27 to 17 min (*p* ≤ 0.001) Figure 1. There was a continued trend toward improvement when allowing for outlier events such as machine breakdown and repair.

**FIGURE 2:**
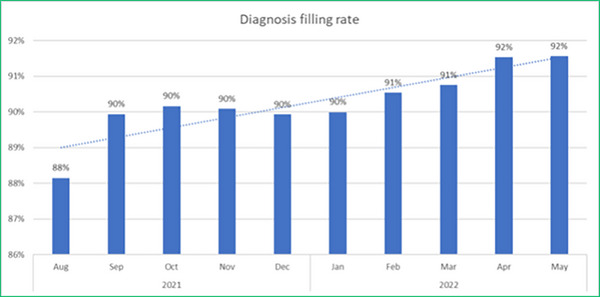
Diagnosis entered for all patients over 10‐month period.

Prior to engagement, no priority was given to data and reporting such as tracking patient demographics and diagnosis. No clear roles or responsibilities were established for data & reporting, and any captured patient information was manually recorded on paper. Within one month of the electronic workflow roll‐out, a 90% diagnosis reporting compliance rate was achieved, further achieving a 92% diagnosis entry compliance average across the period of August 2021 to May 2022 (Figure 2).

With the introduction of robust IT infrastructure, accessibility to the patient EMR system has greatly improved. Additional terminals throughout the department enabled greater access for data recording and digital EMR checks, which is reflected in the reduced patient wait times, as well as the significant data reporting compliance. Additionally, the electronic workflow allowed for remote access for attending Radiation Oncologists to access patient data remotely through internet connection, removing the limitations presented through paper‐based records.

## 4 DISCUSSION

We present here a model of collaboration between a HIC organization and an LMIC cancer care provider with the goal of improving the experience and outcomes of patients treated with radiation. Since initial engagement between GCS and FCC, there have been improvements in treatment plan complexity, as seen in the IMRT output rates recorded over the 12‐month engagement period. Patient wait times have significantly decreased due to the introduction of streamlined electronic workflows, and the uptake of data collection and quality of information gathered since the introduction of the electronic workflows has been significant in providing key insights to the serviced patient demographic and disease prevalence. The improvements seen in all action‐planned domains have confirmed a successful partnership between GCS and FCC.

The value of collaboration across this 12‐month engagement period was essential to initially identify the possible areas of improvement and then successfully roll‐out the designed solutions and workflows across to a paperless system. Compliance from clinical staff and team leads meant that the value of each action plan was able to be successfully implemented and potentially replicated in future POCC departments.

The partnership between the GCS and FCC teams has provided insights into the differing department priorities in different income institutions, providing opportunity to share tested experiences to achieve results. It has been well documented, particularly in HICs that improved patient wait times, leads to a better overall patient experience.^14^ With this knowledge in mind, and often routine target output in HICs, the patient workflow domain targeted streamlined processes to provide opportunities to reduce patient wait times. This has been successfully implemented at FCC.

After the success of the initial action plans, future goals from this collaboration aim to expand the data reporting and IT infrastructure domain to extend across multiple POCC sites. Within the first month of the electronic workflow roll‐out at FCC, a 90% compliance of diagnosis entry was achieved, not only does this provide meaningful information for future projection and disease outcome tracking within the department but also highlights active reporting, leads to a data compliance culture. This process introduced across the wider POCC network will enable greater data collection opportunities which will yield influential insights into the patient demographics and disease prevalence within the wider POCC network.

Future focus within the dosimetry domain will prioritize shifting its focus from remote GCS planning support to the local FCC planning room. As part of this collaboration, GCS has delivered virtual dosimetry training and upskilling sessions for on‐site therapists and physicists. This training, supported by comprehensive training materials and practice cases, provides a robust foundation for future team growth and continued high‐quality plan output. The focus on a local dosimetry service will grant FCC greater autonomy and ownership in ensuring the quality care provided to their patients.

Challenges seen throughout the engagement period can primarily be attributed to a lack of face‐to‐face interaction due to the global COVID‐19 pandemic. Implementing new workflows and technology via remote communication was a challenge, coupled with language and cultural limitations. Through the use of clear procedural documentation, including picture diagrams and practice examples for each new workflow or procedure, we were able to overcome language barriers. Similarly, by maintaining constant virtual communication and offering flexibility with meeting times, we successfully overcame cultural barriers. Another challenge was attributed to lack of baseline data. As prior to the shift to an electronic workflow as seen through this project, data such as patient wait times as well as IMRT output were all based on approximations from paper records. Within the next 12‐month data collection period we expect to obtain greater insight into department operations as we develop larger data pools to identify trends and additional improvement points. Despite these limitations, this initiative has demonstrated a viable model of collaboration between a HIC and LMIC center to improve radiation care delivery processes geared toward improving patient access to quality care.

## 5 CONCLUSION

We present a model of collaboration between a LMIC and a HIC institution to improve radiation delivery and access. Through the GCS and FCC partnership, the implementation of streamlined electronic workflows has significantly reduced overall patient wait times, resulting in notable efficiency gains. Similarly, the integration of data collection points throughout the workflow enhances safe and robust data collection, empowering the department for informed decision‐making, ultimately ensuring greater accessibility to radiation therapy services.

## AUTHOR CONTRIBUTIONS

Diana NG conceived and designed the study. Tatiana De Martin collected and analyzed the data and wrote the manuscript. Po Ting Wu and Peter Huang assisted with data collection, contributed to the data analysis, and provided critical revisions to the manuscript. Jennifer Davis and Jamil Serdina provided expertise in the study design. Jamie Nash, Sarah Knighton, Sherwin Cala, Tisha Lee V. Eduave, and Anthony Albert Abad provided guidance and supervision throughout the project, reviewed and edited the manuscript, and provided intellectual input. All authors reviewed and approved the final version of the manuscript.

## CONFLICT OF INTEREST STATEMENT

GenesisCare Solutions functions as a service provider in the context of this initiative, offering essential services to meet the project objectives. The financial funding GCS receives is contingent upon the successful delivery of these services. POCC was an existing customer of GenesisCare Solutions.

## ETHICS STATEMENT

This paper does not require ethical approval as it does not involve sensitive data that would necessitate ethical review.
